# Evaluation of the host immune response assay SeptiCyte RAPID for potential triage of COVID-19 patients

**DOI:** 10.1038/s41598-023-28178-y

**Published:** 2023-01-18

**Authors:** Maria Milagro Montero, Max Hardy-Werbin, Soledad Gonzalez-Gallardo, Erica Torres, Rebeca Rueda, Irene Hannet, James T. Kirk, Thomas D. Yager, Krupa Navalkar, Maria del Mar Arenas, Itziar Arietta-Aldea, Silvia Castañeda, Joan Gómez-Junyent, Silvia Gómez-Zorrilla, Roberto Guerri-Fernandez, Francisca Sanchez-Martinez, Immaculada López-Montesinos, Ivan Pelegrín, Elena Sendra, Luisa Sorlí, Judith Villar-García, Beatriz Bellosillo, Juan Pablo Horcajada

**Affiliations:** 1 Infectious Disease Department, Hospital del Mar, Barcelona, Spain; 2 IMIM (Hospital del Mar Medical Research Institute), Barcelona, Spain; 3 Department of Medicine and Life Sciences (MELIS), Universitat Pompeu Fabra Barcelona, 08002 Barcelona, Spain; 4 CIBER of Infectious Diseases, Institute of Health Carlos III, Madrid, Spain; 5 Emergency Department, Hospital del Mar, Barcelona, Spain; 6 Pathology Department, Hospital del Mar, Barcelona, Spain; 7 Immunexpress, Seattle, USA

**Keywords:** Infectious-disease diagnostics, Diagnostic markers, Viral infection

## Abstract

Tools for the evaluation of COVID-19 severity would help clinicians with triage decisions, especially the decision whether to admit to ICU. The aim of this study was to evaluate SeptiCyte RAPID, a host immune response assay (Immunexpress, Seattle USA) as a triaging tool for COVID-19 patients requiring hospitalization and potentially ICU care. SeptiCyte RAPID employs a host gene expression signature consisting of the ratio of expression levels of two immune related mRNAs, PLA2G7 and PLAC8, measured from whole blood samples. Blood samples from 146 adult SARS-CoV-2 (+) patients were collected within 48 h of hospital admission in PAXgene blood RNA tubes at Hospital del Mar, Barcelona, Spain, between July 28th and December 1st, 2020. Data on demographics, vital signs, clinical chemistry parameters, radiology, interventions, and SeptiCyte RAPID were collected and analyzed with bioinformatics methods. The performance of SeptiCyte RAPID for COVID-19 severity assessment and ICU admission was evaluated, relative to the comparator of retrospective clinical assessment by the Hospital del Mar clinical care team. In conclusion, SeptiCyte RAPID was able to stratify COVID-19 cases according to clinical severity: critical vs. mild (AUC = 0.93, *p* < 0.0001), critical vs. moderate (AUC = 0.77, *p* = 0.002), severe vs. mild (AUC = 0.85, *p* = 0.0003), severe vs. moderate (AUC = 0.63, *p* = 0.05). This discrimination was significantly better (by AUC or *p*-value) than could be achieved by CRP, lactate, creatine, IL-6, or D-dimer. Some of the critical or severe cases had “early” blood draws (before ICU admission; n = 33). For these cases, when compared to moderate and mild cases not in ICU (n = 37), SeptiCyte RAPID had AUC = 0.78 (*p* = 0.00012). In conclusion, SeptiCyte RAPID was able to stratify COVID-19 cases according to clinical severity as defined by the WHO COVID-19 Clinical Management Living Guidance of January 25th, 2021. Measurements taken early (before a patient is considered for ICU admission) suggest that high SeptiScores could aid in predicting the need for later ICU admission.

## Introduction

In a large meta-analysis of 24,983 patients with COVID-19, Abate et al.^1^ reported that approximately one third of these patients were admitted to the ICU and 39% of those patients died. Those patients that progress to severe disease have been shown to develop a dysregulated immune response and a maladaptive cytokine release^2^. This leads to multiple complications including acute respiratory distress syndrome (ARDS), myocarditis, acute kidney and liver failure and coagulopathy^3^. The similarity of this disease progression to that of another serious disease seen in the ICU—sepsis—has been noted by critical care physicians around the world [e.g. ref.^4^]. Beltran-Garcia et al.^5^ and Gu et al.^6^ reviewed the common features of COVID-19 and sepsis and discussed the possible use of anti-inflammatory therapeutics in the treatment of SARS-CoV-2 disease.

Sepsis is a complex immuno-pathological disorder characterized by an acute pro-inflammatory response that is typically followed by a more chronic anti-inflammatory, immunosuppressive state^7–9^. An early, uncontrollable excessive or hyper-inflammatory response can be associated with septic shock, then cardiovascular collapse, metabolic derangements, and multiple organ dysfunction, and finally death within a few days of onset of sepsis. Until recently, sepsis has been clinically defined as “the presence—probable or documented—of infection together with systemic manifestations of infection”^10,11^. More recently, the definitions and clinical criteria of sepsis and septic shock have changed under Sepsis-3^12,13^. Sepsis-3 now defines sepsis as “a life-threatening organ dysfunction caused by a dysregulated host immune response to infection”. Therefore, the key to diagnosing sepsis according to the new definition lies in determining whether the host has suffered organ dysfunction attributable to a dysregulated immune response, and whether that response is due to an infection.

SeptiCyte® RAPID (Immunexpress, Seattle, WA) is a gene expression assay using reverse transcription—quantitative polymerase chain reaction (RT-qPCR) to measure the relative expression levels of two host response genes, *pla2g7* and *plac8*, that are indicative of a dysregulated immune response during sepsis. The test, used with whole blood samples collected in PAXgene® Blood RNA tubes (Qiagen, Hilden, Germany) and implemented entirely in a single-use cartridge, is a streamlined and fully automated version^14^ of an earlier predicate central laboratory test, SeptiCyte LAB^15^. SeptiCyte RAPID is used in conjunction with clinical assessments, vital signs and laboratory findings as an aid to differentiate infection-positive systemic inflammation (sepsis) from infection-negative systemic inflammation (SIRS) in patients suspected of sepsis. SeptiCyte RAPID generates a quantitative score (SeptiScore®) that increases with increasing likelihood of sepsis.

Analysis of public NGS (next generation sequencing) datasets [e.g. ref.^16^] indicated that SeptiCyte RAPID could discriminate between milder and more severe COVID-19 cases, where the severe COVID-19 cases can be considered examples of viral sepsis. Here, we report on a research study that sought to evaluate the SeptiCyte RAPID assay as a triaging tool for COVID-19 patients requiring hospitalization and potentially ICU care. We find that out study supports the use of SeptiCyte RAPID in the risk stratification of COVID-19 patients, as measured by clinical severity assessment based on final clinical discharge diagnosis, WHO severity score and/or ICU admission. ICU admission is indicated by scores of 6–9 on the WHO 10-point severity scale^17^. We note that part of this work has been published previously in abstract form^18^.

## Materials and methods

### Patients

Patient samples were retrospectively obtained under an Ethics Committee approved protocol (see below) as part of a cohort collected at Hospital del Mar, Barcelona, Spain, between July 28th and December 1st, 2020. Patients admitted to the hospital with clinical signs of COVID-19 illness were eligible. SARS-CoV-2 infection was confirmed by RT-qPCR (Roche cobas® or Abbott Real-time SARS-CoV2 Assay®) or antigen testing (Abbott Binax NOW™ rapid SARS-CoV-2 antigen assay), depending on test availability. Samples used for Septicyte RAPID testing were obtained from patients that were not receiving antibiotic treatment and had been obtained in the first 48 h after arrival to the hospital.

### Ethics committee approval

The study was designed to use biospecimens and associated clinical/demographic/ laboratory patient information already collected for the purpose of performing clinical COVID-19 research by Hospital del Mar. Ethics committee approval was obtained from the Parc de Salut Mar, Barcelona, Spain on November 27th, 2020 (Nr. 2020/9489/I; serial number IDCES-5331405), stating that the study would be performed by the Infectious Diseases and Pathology Hospital del Mar services. The study was conducted in accordance with the relevant institutional guidelines and regulations, and informed consent was obtained from all patients or legal representatives for the use of their samples and associated demographic and clinical information.

### Clinical, laboratory, and demographic data

Clinical data were collected during patient standard of care and final discharge diagnosis. Measurements of white blood cell (WBC), neutrophil and lymphocyte counts, lactate, creatine, C-reactive protein (CRP), IL-6 and D-dimer levels were performed within 24 h of hospital admission. SpO2 measurements were performed within 72 h of hospital admission, as per hospital protocol. COVID-19 disease severity assessments were provided by the clinical care team at Hospital del Mar. Supplementary Table 1 presents a list of clinical variables specified in the case report form (CRF) which were collected depending on availability, to help with assessment of clinical disease severity and diagnostic classification by the clinical care team. All clinical outcome parameters were recorded by the Hospital Del Mar clinical care team.

### SeptiCyte RAPID assays

Peripheral blood samples were collected in PAXgene blood RNA tubes within 48 h of hospital admission, and were frozen at − 70 to − 80 °C according to the manufacturer’s instructions. Testing of banked PAXgene blood RNA samples from eligible patients was performed at Hospital del Mar by qualified personnel, on qualified Biocartis Idylla™ systems installed with the SeptiCyte RAPID assay software. The PAXgene blood RNA samples were thawed and gently inverted 10 × to ensure the tube contents were homogeneous. A 0.9 mL aliquot was pipetted into the SeptiCyte RAPID cartridge, and run as per SeptiCyte RAPID Instructions for Use. Data collection and analysis to produce SeptiCyte RAPID scores were automatically conducted by the Idylla system.

### SeptiCyte RAPID performance evaluation

SeptiCyte RAPID performance was evaluated by comparison to a ‘gold standard’ consisting of retrospective clinical diagnosis by the clinical care team at Hospital del Mar. The retrospective diagnosis employed the COVID-19 clinical severity categories (Table 1) defined in^19^, the WHO COVID-19 Clinical Management Living Guidance of January 25th, 2021 (hereafter called “WHO COVID-19 Guidance 2021”).

**Table 1:** Patients in the study cohort, as classified by the Hospital del Mar clinical care team.

COVID severity category	Description
Mild (n = 10)	The clinical symptoms are mild with no pneumonia manifestations found in imaging
Moderate (n = 27)	Patients have symptoms such as fever and respiratory tract symptoms with pneumonia manifestations seen on imaging
Severe (n = 70)	Any of the following criteria: respiratory rate ≥ 30 breaths/min; oxygen saturation ≤ 93% in resting state; arterial partial pressure of oxygen (PaO2)/oxygen concentration (FiO2) ≤ 300 mm Hg. Patients with > 50% lesions progression within 24 to 48 h in lung imaging should be treated as severe cases
Critical (n = 39)	Patients meeting any of the following criteria: occurrence of respiratory failure requiring mechanical ventilation; presence of shock; other organ failure that requires monitoring and treatment in the ICU

The WHO COVID-19 Guidance 2021^19^ was used to define the COVID severity categories. There were a total of 146 patients in the study.

### Statistical and bioinformatics analyses

Receiver operating characteristic (ROC) curves were calculated using the pROC package^20^ in the R programming language^21^ and verified with the JROCFIT applet^22^ available at www.rad.jhmi.edu/jeng/javarad/roc/JROCFITi.html. Area under curve (AUC) was used as a performance metric. Differences between the AUCs of two ROC curves were evaluated by resampling as described by^23^. Principal Component Analysis was performed using the FactoMineR package in R^24^. Kolmogorov–Smirnov tests, for comparing a variable’s distributions in 2 populations, were conducted using the online applet at http://www.physics.csbsju.edu/stats/KS-test.html. Welch t-tests, for comparing means between two independent groups without assuming equal population variances, were conducted with the Stats package in R^25^. For one-way ANOVA a custom R script was written to use the "aov" function in the "stats" R package. Multivariable regression used the online applet at: www.statskingdom.com/410multi_linear_regression.html.

## Results

### Demographic and clinical characteristics

The study cohort comprised 146 patients for whom blood samples were taken within 48 h of hospital admission. The cohort consisted of a heterogeneous group of non-acute and acutely ill patients with varying phases and degrees of inflammatory response, as well as a range of comorbidities. Of these patients, 39 had COVID-19 severity assignments of “critical”, 70 had assignments of “severe”, 27 were assigned as “moderate” and 10 as “mild” by the criteria of Table 1. Of the 146 patients in the cohort, 43 (29%) were admitted to ICU and of these, 10 (23%) were classified as severe and 33 (77%) were classified as critical. Table 2 summarizes the demographic and clinical characteristics of the patients in the study cohort, stratified by their severity assignments as adjudicated by the clinical care team at Hospital del Mar. Supplementary Table 2 summarizes the interventions provided to the patients in the cohort. The severe and critical patients required more interventions to manage their clinical trajectories as compared to the moderate and mild cases. Hospital del Mar had a well-established protocol in place involving the early administration of corticosteroids.

**Table 2:** Demographic and Clinical characteristics of patients stratified by COVID severity.

Parameter	Mild (n = 10)	Moderate (n = 27)	Severe (n = 70)	Critical (n = 39)	Critical & Died (n = 13)	*p*-value
Age	76 (64–77)	59 (50–66)	64 (51–79)	69 (62–78)	79 (66–84)	0.002
Sex (M/F)	4/6	14/13	35/35	26/13	9/4	0.3
Race / ethnicity	White = 8 Asian = 1, Unknown = 1	White = 21 Asian = 2 Unknown = 4	White = 49 Asian = 12 Black = 1 Unknown = 8	White = 24 Asian = 9 Black = 1 Unknown = 5	White = 9 Asian = 3 Black = 1	0.41
% SpO_2_ min	94 (93.25–95)	94 (94–95)	91 (88–93)	89 (86–91)	88 (86–91)	< 0.001
Respiratory Rate, breaths/min (max)	23 (18–26)	22 (20–24)	28 (22–32)	28 (24–32)	28 (24–32)	< 0.001
WBC (cells/mm^3^)	5540 (4712–6530)	6090 (4995–7380)	6230 (4900–7360)	6690 (5250–8715)	8700 (6830–8900)	0.5
Neutrophil/Lymphocyte ratio	4.9 (2.6–5.9)	3.1 (2.5–5.0)	4.3 (2.8–6.5)	6.1 (3.8–9.5)	8.9 (5.6–12.0)	0.004
Lactate (mmol/L)	1.57 (1.23–2.43)	1.23 (1.07–1.45)	1.4 (1.07–1.7)	1.54 (1.21–2.04)	1.66 (1.47–2.36)	0.009
Creatinine (mg/dl)	1.22 (0.90–1.85)	0.81 (0.69–0.95)	0.85 (0.7–1.06)	1.08 (0.88–1.42)	1.31 (1.10–2.37)	0.005
CRP (mg/L)	6 (0–7)	5 (3–8)	7 (3–10)	9 (5–14)	7 (5–14)	0.020
IL-6 (pg/ml)	16.7*	35 (21–44)	29 (7–51)	37 (15–84)	40 (33–73)	0.7
D-dimer (mg/L)	890 (710–1025)	590 (420–760)	645 (415–1128)	720 (430–1015)	2530 (735–7642)	0.053
SeptiScore	4.9 (4.5–6.1)	6.0 (5.8–6.8)	6.8 (5.9–7.7)	7.1 (6.6–8.0)	7.1 (6.7–8.3)	< 0.001

The severity categories were defined according to the WHO COVID-19 Guidance 2021^19^. There were 146 patients in this cohort. Recorded values are medians (interquartile ranges). *p*-values were calculated with one-way ANOVA. [*IL-6 was measured for only one Mild patient, so IQR could not be calculated.]

### Principal component analysis

**Figure 1:**
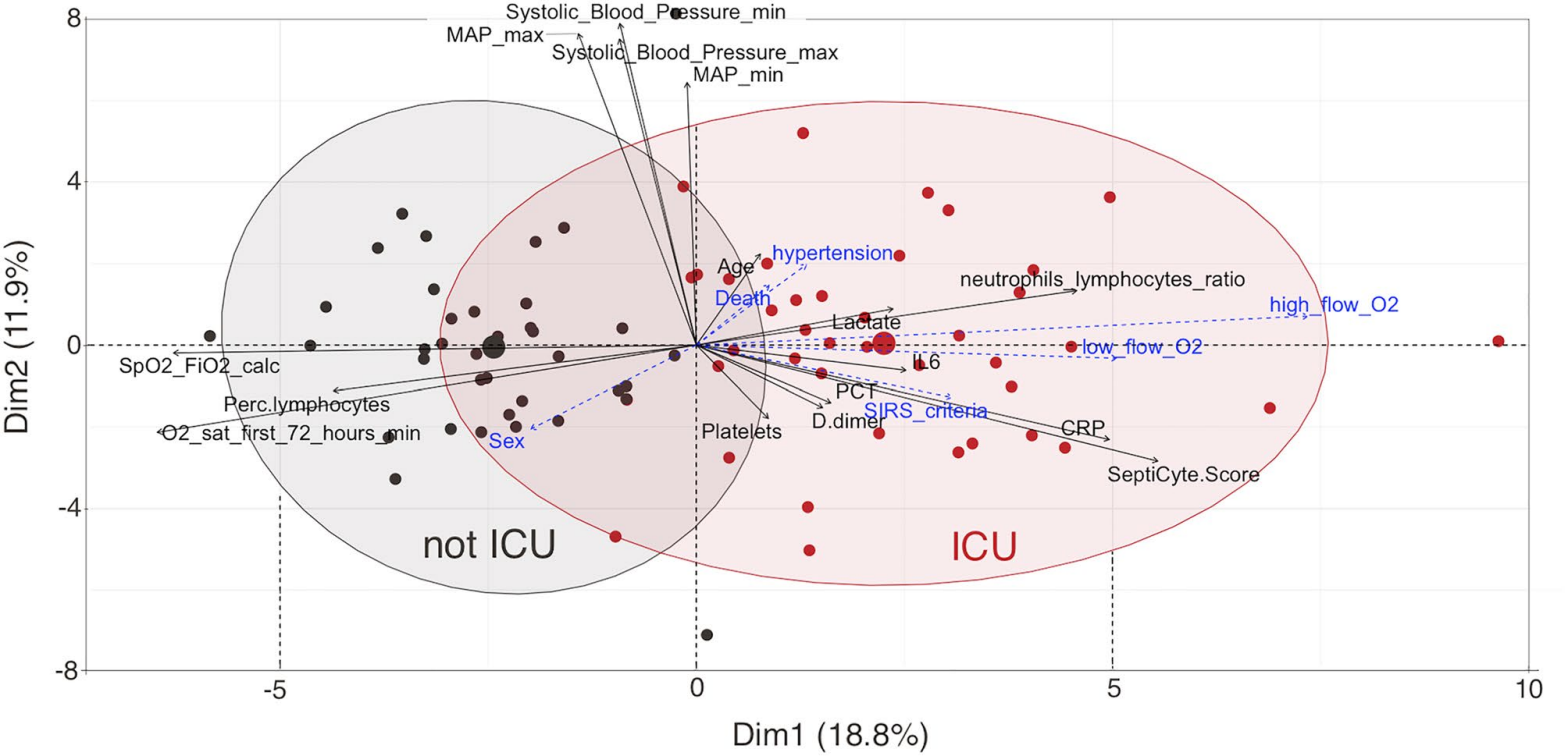
Principal Component Analysis (PCA) biplot, for separation of patients with ICU admission vs. non-admission. A total of **45** variables were used to define the dimensions (“eigenvectors”) of the PCA plot. A total of n = 77 patients were analyzed, representing critical and severe COVID cases that needed ICU admission (red points; N = 40) vs. mild and moderate cases that did not need ICU admission (black points; N = 37). These were the two maximally different groups within the study cohort. The variables shown with blue dotted arrows are supplementary continuous variables which did not contribute to the construction of the PCA dimensions, but which are presented to aid in the interpretation in the PCA biplot**.** Additional details are provided in Supplementary Table 3.

Principal Component Analysis (PCA)^26^ is a powerful tool to help visualize information contained in complex datasets that are highly multidimensional, and to identify key variables. We conducted a PCA in which ICU admission vs. non-admission was the variable chosen as the basis for class separation. A total of n = 77 patients were analyzed, representing the two groups within the study cohort that differed to the greatest extent: critical and severe COVID cases that needed ICU admission (N = 40) vs. mild and moderate cases that did not need ICU admission (N = 37). The remaining 69 patients in the cohort, who were adjudicated as severe or critical COVID cases but not admitted to ICU, were not used in the PCA analysis, because the analysis was intended to probe the differences between the two maximally different groups within the study cohort. A list of 45 quantitative variables used to define the dimensions of the PCA is given in Supplementary Table 3. Figure 1 shows a “biplot” which overlays the individual patients (represented by dots) with vectors (represented by arrows) which indicate the direction and strength of contributions of different single variables to the class separation along PCA dimensions 1 and 2. The first two dimensions of the PCA explain 30.7% of the variance in data. The length of each arrow from the origin is proportional to the contribution of its variable to the construction of the given dimension. When the angle between two arrows is small, the associated variables are highly correlated: for example, SeptiScore is correlated with SeptiCyte Band as would be expected, and is also correlated with other clinical variables that are elevated in the sepsis phenotype or in patients admitted to the ICU, such as C-reactive protein (CRP), D-dimer, interleukin-6 (IL-6), procalcitonin (PCT), and indicators of the systemic inflammatory response syndrome (SIRS). When the angle between arrows is at approximately 90 degrees there is no correlation between the variables, as can be seen e.g. in the comparison of lymphocyte/neutrophil ratio and blood pressure.

The PCA analysis provides evidence that patients admitted to the ICU have a higher SeptiScore compared to patients that were not admitted to the ICU; and that SeptiScore contributes more than other parameters such as D-dimer, IL-6, and CRP toward the construction of the principal components for identifying patients that needed ICU admission.

We also conducted an additional PCA in which a third group, consisting of the 69 critical/severe, non-ICU patients was added. This additional analysis is presented as Supplementary Fig. S1. The addition of this third group does not change the conclusions drawn from the initial PCA: the directions and lengths of the individual vectors are largely unchanged.

### Stratification by clinical severity

**Figure 2:**
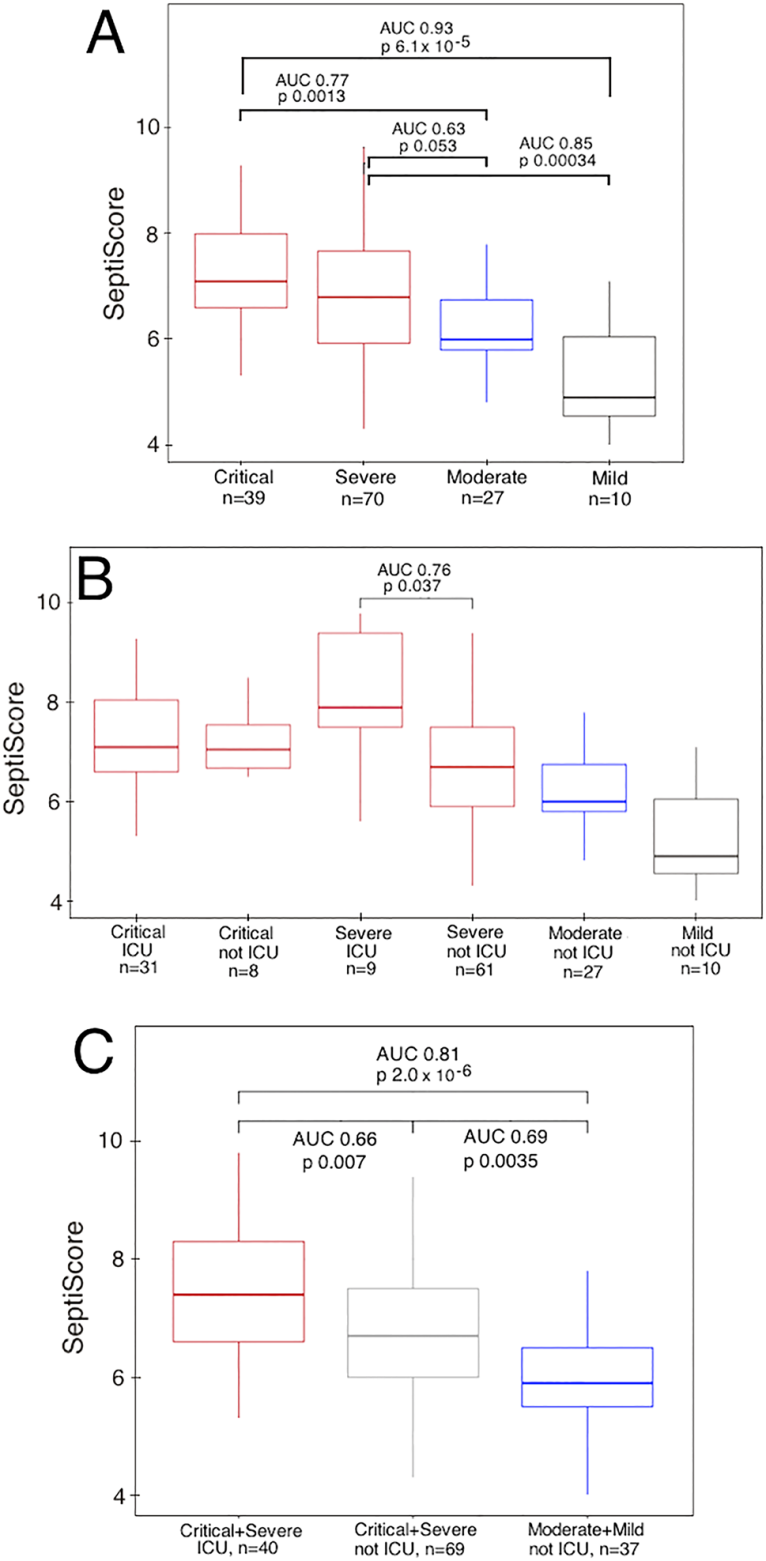
**(A)** SeptiCyte RAPID scores across the COVID-19 severity categories defined in the WHO COVID-19 Guidance 2021^19^, as assessed by the clinical care team at Hospital del Mar (n = 146). **(B)** SeptiCyte RAPID scores across the COVID-19 severity categories, after further stratification by ICU admission vs. non-admission. **(C)** SeptiCyte RAPID scores for differentiating COVID patients that needed ICU admission from the cases that did not need ICU admission.

Figure 2A presents the distribution of SeptiScores across the four COVID-19 clinical severity categories defined in the WHO COVID-19 Guidance 2021^19^, using the assessments provided by the care team at Hospital del Mar. This figure shows that the median SeptiScores were significantly higher for the critical and severe groups than for the moderate and mild groups.

A further stratification analysis by hospital location (ICU vs. non-ICU) is presented in Fig. 2B, in which SeptiScores are stratified based on whether or not the patients were admitted to the ICU (in addition to their COVID severities). It was found that relatively few critical cases (8/41 = 19.5%) were managed outside the ICU. While the SeptiScores of the critical group do not appear to differ between ICU vs. non-ICU locations, the SeptiScores of the severe group are significantly higher for patients in ICU (n = 9) vs. patients not in ICU (n = 61) with AUC 0.76 and *p*-value 0.026 for this comparison.

As indicated in Fig. 2C, if the critical and severe patients who were admitted to the ICU (red, n = 40) are compared to the mild and moderate cases none of whom were admitted to the ICU (blue, n = 37), the performance of SeptiCyte RAPID has an AUC of 0.80 and *p*-value of 2.48 × 10^–6^. Also, the median SeptiScores of critical or severe COVID cases that were not admitted to the ICU (grey, n = 69) are lower than those admitted to the ICU (red, n = 40), but higher than the mild or moderate cases not admitted to the ICU (blue, n = 37).

A ROC test comparison (Table 3) indicated that SeptiCyte RAPID was significantly better at distinguishing the critical + severe ICU patients from the moderate + mild non-ICU patients, as compared to similarly timed D-dimer, lactate, IL-6 or creatinine measurements which are typically used to assess the severity of patient clinical trajectories. The AUC of SeptiCyte RAPID (AUC = 0.81) was also found to be higher than that of CRP (AUC = 0.67), although this comparison was on the edge of statistical significance (*p*-value = 0.067 by bootstrap resampling).

**Table 3:** Performance of SeptiCyte RAPID relative to other clinical laboratory tests.

Lab Test	1. Critical + Severe in ICU (N = 40)	2. Critical + Severe not in ICU (N = 69)	3. Moderate + Mild not in ICU (n = 37)	1. versus 2. AUC ± SE (*p*-value)	1. versus 3. AUC ± SE (*p*-value)
SeptiCyte RAPID	n = 40 (100%)	n = 69 (100%)	n = 37 (100%)	0.66 ± 0.05	0.81 ± 0.050
CRP	n = 39 (97.5%)	n = 67 (97.1%)	n = 36 (90%)	0.58 ± 0.06 (0.22)	0.67 ± 0.063 (0.067)
Lactate	n = 35 (87.5%)	n = 51 (73.9%)	n = 28 (70%)	0.56 ± 0.07 (0.08)	0.62 ± 0.073 (0.024)
Creatinine	n = 40 (100%)	n = 68 (98.5%)	n = 37 (100%)	0.56 ± 0.06 (0.25)	0.60 ± 0.065 (0.009)
IL-6	n = 30 (75%)	n = 32 (46.4%)	n = 15 (37.5%)	0.59 ± 0.07 (0.74)	0.56 ± 0.087 (0.012)
D-dimer	n = 38 (95%)	n = 65 (94.2%)	n = 32 (80%)	0.56 ± 0.06 (0.29)	0.50 ± 0.070 (0.0004)

Critical + severe cases in ICU (n = 40) were compared to moderate + mild cases not in ICU (n = 37). Values in parentheses ( ) represent the % of patients in each clinical category for whom the relevant laboratory test results were available. There were some missing values as indicated by the percentages in the table. AUC values were compared to the SeptiCyte RAPID AUC with a bootstrap method as described in^23^, and *p*-values for the AUC comparisons are indicated.

### Comparative performance of SeptiCyte RAPID and IL-6

**Figure 3:**
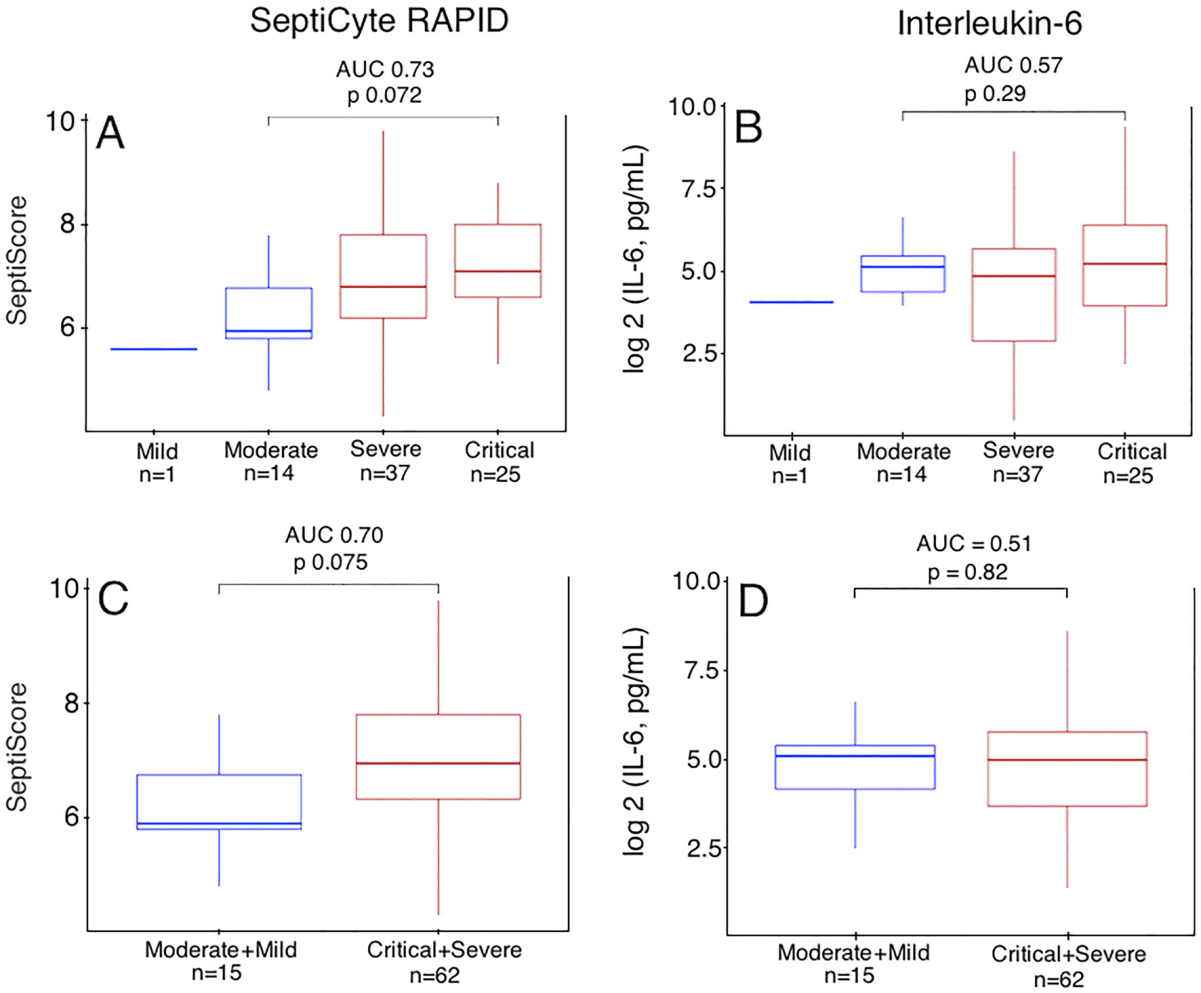
Performance of SeptiCyte RAPID relative to IL-6, in discriminating COVID severities. Panel A: distribution of SeptiScores across the different COVID severity classes (n = 77; only those patients who also had IL-6 measurements were considered). Panel B: IL-6 levels in pg/ml, log2 transformed, for patients across the same severity classes (n = 77; all available data). Panel C: SeptiCyte RAPID for mild + moderate grouped together, vs. critical + severe grouped together. Panel D: IL-6 for mild + moderate grouped together, vs. critical + severe grouped together.

Although it is not current practice at the Hospital del Mar study center, IL-6 levels above 35 pg/ml have been used elsewhere to ascertain the need for mechanical ventilation in COVID-19 patients^27^. Figure 3 shows the performance of SeptiCyte RAPID relative to IL-6 in a subset of 77 patients for whom both values were measured within 24 h of hospital admission. Panels A, C show the distribution of SeptiScores across varying COVID-19 severities, whereas Panels B, D show the IL-6 levels in pg/ml for patients across the same clinical severities.

The performance of SeptiCyte RAPID for distinguishing critical (n = 25) from moderate (n = 14) COVID cases (Panel A) has an AUC of 0.73 (t-test *p* = 0.06) and a Kolmogorov–Smirnov D value of 0.528 (*p* = 0.007). In contrast, the same discrimination with IL-6 (Panel B) has AUC 0.55 (t-test *p* = 0.3) and a non-significant Kolmogorov–Smirnov D value of 0.275 (*p* = 0.43).

If moderate + mild (n = 15) cases are grouped together, and similarly for critical + severe cases (n = 62), then the performance of SeptiCyte RAPID for distinguishing between these composite groups (Panel C) has AUC of 0.70 (t-test *p* = 0.066), with a Kolmogorov–Smirnov D value 0.495 (*p* = 0.003). In contrast, the performance of IL-6 (Panel D) is AUC 0.51 (t-test *p* = 0.8), with a non-significant Kolmogorov–Smirnov D value of 0.265 (*p* = 0.33).

It is known that IL-6 levels can be modulated by corticosteroid treatment^28–30^ whereas SeptiCyte RAPID appears unaffected^31^. As the Hospital del Mar patients generally were treated with corticosteroids early during their transit through hospital, this might provide an explanation for the absence of diagnostic power for IL-6, as opposed to SeptiCyte RAPID, in this study cohort. Further consideration of this point is presented in the Discussion.

### Predicting ICU admission

**Figure 4:**
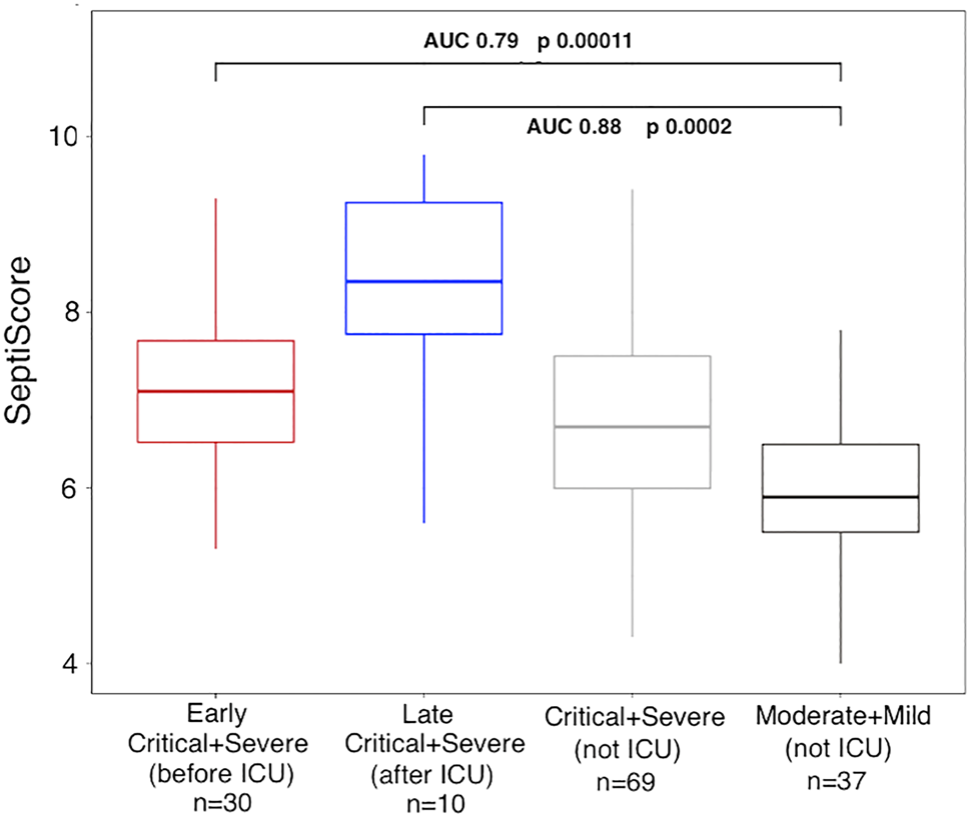
Predicting ICU admission using SeptiCyte RAPID (n = 146).

Figure 4 provides evidence that SeptiCyte RAPID could potentially be used to predict the need for ICU admission. Some of the critical and severe cases had “early” blood draws (i.e. before ICU admission; red, n = 30). When these were compared to moderate and mild cases not in ICU (black, n = 37), SeptiCyte RAPID had an AUC of 0.78 (*p* = 0.00012). This comparison suggests that a high SeptiScore, measured early (before a patient is considered for ICU admission), might predict the need for later ICU admission.

For reference, there were also critical or severe cases which had “late” blood draws (i.e. after ICU admission; blue, n = 10). When these were compared to moderate and mild cases not in ICU (black, n = 37), SeptiCyte RAPID had an AUC of 0.88 (*p* = 0.002). Here, a high SeptiScore measured after a patient was already in ICU most likely describes the patient’s current state, as opposed to being a predictor of a future state. In these ICU patients, a high SeptiScore indicated a sepsis phenotype, including caused by either a viral or bacterial infection.

### SeptiCyte RAPID in relation to oxygen therapy

In two exploratory analyses, we assessed the correlation between elevated SeptiScores and the need for enhanced oxygen therapy during a patient’s clinical trajectory. We considered therapeutic escalation along the following axis: no oxygen needed, low flow oxygen therapy, high flow oxygen therapy, mechanical ventilation (MV), mechanical ventilation + intubation (MV + i), and extracorporeal membrane oxygenation (ECMO). Progression along this axis would indicate a deteriorating clinical trajectory.

**Figure 5:**
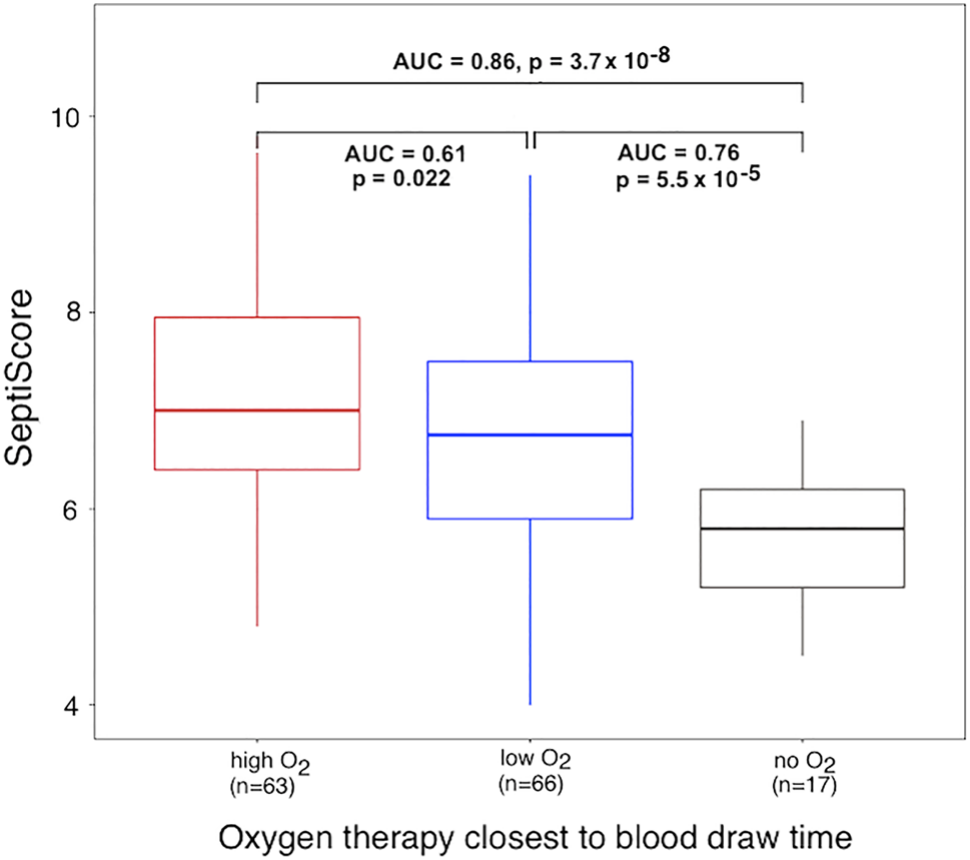
SeptiScore stratified by type of oxygen therapy closest to blood draw. A total of 146 patients are represented in this plot.

In a first analysis, we asked whether elevated SeptiCyte RAPID scores correlated with the need for high flow oxygen therapy. This level of therapy falls between no/low flow oxygen, and the more extreme therapies involving mechanical ventilation, intubation, or ECMO. Figure 5 compares the group needing high flow oxygen (red, n = 63) to those patients not needing supplemental oxygen (black, n = 17). The performance of SeptiCyte RAPID in this comparison had AUC 0.86 (*p* = 3.7 × 10^–8^). The AUC between the groups that needed high flow (red, n = 63) vs. low flow oxygen (blue, n = 66) was 0.61 (*p* = 0.022). Finally, the AUC between the groups that needed low flow oxygen (blue, n = 66) vs. those that did not need oxygen supplementation (black, n = 17) was 0.76 (*p* = 5.5 × 10^–5^).

In a second analysis, we conducted a multivariable linear regression to explore whether the SeptiCyte RAPID score, in combination with other clinical parameters that typically would be measured early during a patient’s transit through the hospital, correlated with the degree of oxygen therapy administered. We represented the extent of need for oxygen therapy by a “weighted oxygen therapy index” (Y) defined by the following formula:

1 $$ \begin{aligned} {\text{Y}} & = 1*\left( {{\text{days}}\;{\text{low}} - {\text{flow}}\;{\text{O}}_{2} } \right) + 2* \left( {\# \;{\text{days}}\;{\text{high}} - {\text{flow}}\;{\text{O}}_{2} } \right) + 3*\left( {\# \;{\text{days}}\;{\text{non}} - {\text{invasive}}\;{\text{MV}}} \right) \\ & \quad + 4*\left( {\# \;{\text{days}}\;{\text{MV}} + {\text{intubation}}} \right) + 5*\left( {\# {\text{days}}\;{\text{ECMO}}} \right) \\ \end{aligned} $$

Thus, as the oxygen therapy became increasingly extreme or invasive, or drawn out in duration, it was weighted more heavily. The clinical input variables {Xi} that were considered were among those which typically would be obtained upon hospital admission or shortly thereafter, that related to COVID-19 disease presentation, and that had few if any missing data in our dataset: X_1_ = age, X_2_ = hypertension, X_3_ = SeptiScore, X_4_ = WBC count, X_5_ = neutrophils, X_6_ = lymphocytes, X_7_ = CRP, X_8_ = D-dimer, X_9_ = SpO_2_ max (within 72 h).

A linear regression analysis Y = f{Xi} returned a significant fit (right-tailed, F(1,32) = 7.429, *p*-value = 0.0022) to the following model:

2 $$ {\text{Y}} = - 123.45 + 13.76*{\text{X}}_{3} + 0.0088*{\text{X}}_{4} $$

Since *p*-value < α (0.05), we reject the null hypothesis H_0_ and conclude that SeptiScore (X_3_) and WBC count (X_4_) appear to be correlated with the need for extreme oxygen therapy in this cohort.

We note that the SeptiScore measurements were taken on either side of the time when the oxygen therapy was first administered. Because of this timing variability, it was not possible to determine if SeptiScore had predictive capabilities, with respect to the need for escalatory oxygen therapy. Thus, in these analyses, SeptiScore is most likely indicative of the current state of patients, rather than predictive of future states.

### Discriminating between discharge and death

Finally, we examined whether an early SeptiCyte RAPID measurement could discriminate between patients who ultimately were discharged or who died. A modest predictive ability (AUC 0.70, *p* = 0.02) was observed. In the 13 patients that died, SeptiScore was measured between 1 and 52 days prior to their death. In the 133 patients that were discharged, a SeptiScore was drawn between 0 and 55 days prior to their hospital discharge. A precise interpretation of these results may be difficult, however, because of the long time gaps that occurred in some cases between blood draw and the event of interest (discharge or death).

## Discussion

We report on a study using a novel host immune response test, SeptiCyte RAPID, in COVID-19 confirmed patients who required hospitalization during a heavy case load period in a tertiary hospital in Barcelona, Spain. The need for early effective interventions as well as deciding which patients should receive ICU care has been a constant focus of our clinical care team, as it has been and continues to be in many places around the world.

Although certain patient characteristics may be helpful—for example, our data confirms a well-reported higher rate of critical/severe disease with increasing age—certain information may only become available hours or even days after hospitalization. Also, a vast intake requirement of patients may be overwhelming, especially for more junior staff or less-resourced settings. Based on worldwide research as well as our own findings in this disease where critical or severe cases are commonly found to progress to sepsis, early on Hospital del Mar implemented an anti-inflammatory intervention protocol that was fully adopted by the clinical care team (see Supplementary Table 2).

One of the earliest interventions for most COVID cases was the provision of low flow oxygen, to compensate for incipient organ dysfunction in the respiratory system. When comparing patients who did not need supplemental oxygen with those who required low flow oxygen, the discrimination by SeptiCyte RAPID had an AUC of 0.75 (Fig. 5). If patients did not improve on low flow oxygen, additional interventions were offered in the form of higher flow oxygen and progressively more invasive interventions such as mechanical ventilation, mechanical ventilation with intubation, and ECMO. A comparison between patients not requiring oxygen and those requiring high flow oxygen produced an AUC of 0.87 (Fig. 5). We note, though, that the SeptiScores used in this analysis were taken on either side of the time when the oxygen therapy was first administered. Thus, as far as this analysis is concerned, the SeptiScores could not be used in a predictive sense, and more likely described the patients’ current states, as opposed to being a predictor of their future states.

IL-6 levels above 35 pg/ml have been used to ascertain the need for mechanical ventilation in COVID-19 patients^27^. IL-6 levels are known to be modulated by corticosteroid treatment^28–30^. In a cohort where the majority of the patients were treated early in the clinical trajectory with corticosteroids, clinical lab parameters such as IL-6 were found to become less reliable at distinguishing the critical + severe cases from the moderate + mild cases, or for ascertaining the need for mechanical ventilation using the recommended threshold of 35 pg/ml (Fig. 3). SeptiCyte RAPID is not affected by corticosteroid treatment (Fig. 3 and ref.^31^) making it an effective diagnostic for distinguishing severe or critical cases away from moderate or mild cases regardless of ongoing corticosteroid treatment.

In the more severe or critical cases, COVID-19 typically progresses beyond localized lung damage, and begins to involve multiple organ systems in a state resembling or identical to viral sepsis. Organ dysfunction as it relates to sepsis is a composite metric comprised of the failure of one or more organ systems in the context of uncontrolled inflammation in response to a severe infection. In this analysis, SeptiCyte RAPID may find utility as a biomarker for the organ dysfunction that might be developing in some more extreme cases of COVID-19.

We found that SeptiCyte RAPID, measured early, show some predictive ability in stratifying patients that will vs. will not require future ICU admission (Fig. 4). As SeptiCyte RAPID was originally trained on a cohort of sepsis cases admitted to the ICU^32^, it is not unexpected that this assay can discriminate between ICU vs. non-ICU patients in a similar but previously untested, new infectious disease context, starting with a dysfunctional immunological response to COVID-19 infection.

The tendency of COVID-19 patients to require ICU -level care, and thus the applicability of SeptiCyte RAPID to the COVID-19 scenario described in this paper, might not remain constant in the future, due to multiple factors. There is a wide range of host susceptibility to deleterious immune responses to infection. The proportion of the affected population that carries some immunity to SARS-CoV-2 continues to increase, either through vaccinations or natural infection. On the other hand, SARS-CoV-2 continues to evolve to create variants that exhibit immune-escape capabilities. Continued use of SeptiCyte RAPID in hospital clinics would allow the range of applicability of the test to be evaluated on an ongoing basis.

Our study has several strengths and limitations. Strengths include acquisition of a detailed and complete clinical dataset (over 100 parameters), and tight control over the timing of data collection. Both strength and weakness resulted from the nature of patient enrollment and sample selection in the study. The samples were selected retrospectively from a prospectively collected sample bank, which resulted in a cohort with very few missing parameter values, but on the other hand may have introduced the possibility of bias in sample selection. The study size (n = 146) is of limited statistical power when subgroups of patients are analyzed. Finally the composite endpoint of ICU admission was considered in some analyses, which may have introduced a dependency on site-specific ICU admission criteria.

## Conclusions

We have assessed the ability of SeptiCyte RAPID to stratify patients with respect to COVID-19 severity, and to provide an estimate of risk of ICU admission. SeptiCyte RAPID can differentiate the critical or severe cases of COVID-19 that needed to be admitted to the ICU from the moderate and mild cases that did not, with an AUC of 0.80. SeptiCyte RAPID performed better than CRP, D-dimer, lactate, IL-6 and creatinine for assessing severity of COVID disease, and for distinguishing the critical or severe cases needing admission to ICU from mild and moderate cases not requiring ICU level care. We have also shown that SeptiCyte RAPID can stratify patients according to the need for increasing levels of oxygen therapy. Finally, SeptiCyte RAPID appears to have some ability (AUC 0.70) to predict future patient trajectories resulting in either discharge or death. In light of these findings, we suggest that elevated SeptiCyte RAPID scores could potentially serve as warning indicators that enhanced levels of patient monitoring is called for.

In summary, the performance of SeptiCyte RAPID exceeded other single biomarker assays used to assess COVID severity, and also had some predictive ability for major adverse events and interventions in the clinical patient trajectory. With an availability within ~ 1 h from blood collection and minimal user interaction, the assay may be helpful in stratifying patients at the moment of hospital admission. The study should be expanded to increase the value of this finding.

## Supplementary Information

Supplementary Information.

## Data Availability

The datasets used and/or analysed during the current study available from the corresponding author on reasonable request.

## Abbreviations

ARDS: Acute respiratory distress syndrome
AUC: Area under receiver operating characteristic curve
CE: Conformité Européene
CBC: Complete blood count
COVID-19: Coronavirus disease 2019
CRF: Case report form
CRP: C-reactive protein
ECMO: Extracorporeal membrane oxygenation
FDA: United States Food and Drug Administration
ICU: Intensive care unit
IL6: Interleukin-6
K–S: Kolmogorov–Smirnov
MV: Mechanical ventilation
NGS: Next-generation sequencing
PCA: Principal component analysis
PCT: Procalcitonin
PLA2G7: Gene for phospholipase A2 group VII protein [HGNC:9040]
PLAC8: Gene for placenta associated 8 protein [HGNC:19254]
ROC: Receiver operating characteristic [curve]
RRT: Renal replacement therapy
RT-qPCR: Reverse-transcription quantitative polymerase chain reaction
SIRS: Systemic inflammatory response syndrome
SARS-CoV-2: Severe acute respiratory syndrome coronavirus 2
USA: United States of America
WBC: White blood cell
WHO: World Health Organization
