# Supplementary material for: Evaluation of the host immune response assay SeptiCyte RAPID for potential triage of COVID-19 patients
Source: Sci Rep. 2023 Jan 18;13:944. doi: 10.1038/s41598-023-28178-y (PMC9845827; doi:10.1038/s41598-023-28178-y)
Supplement: Supplementary file 1 — Supplementary Information. [file 41598_2023_28178_MOESM1_ESM.docx]

**Evaluation of the host immune response assay SeptiCyte RAPID for potential triage of COVID-19 patients**

Maria Milagro Montero^1,2,3,4^, Max Hardy-Werbin^2,5^, Soledad Gonzalez Gallardo^2^, Erica Torres^6^, Rebeca Rueda^6^, Irene Hannet^7^, James T Kirk^7^, Thomas D Yager^7^, Krupa Navalkar^7^, Maria del Mar Arenas^1,2^, Itziar Arietta-Aldea^1,2^, Silvia Castañeda^1,2^, Joan Gómez-Junyent^1,2^, Silvia Gómez-Zorrilla^1,2,3,4^, Roberto Guerri-Fernandez^1,2,3,4^, Francisca Sanchez-Martinez^1,3^, Immaculada López-Montesinos^1,2^, Ivan Pelegrín^1,2^, Elena Sendra^1,2^, Luisa Sorlí^1,2,3,4^, Judith Villar-García^1,2,3^, Beatriz Bellosillo^2,6*§^, Juan Pablo Horcajada^1,2,3,4^*

^1^ Infectious Disease Department, Hospital del Mar, Barcelona (Spain)

^2^ IMIM (Hospital del Mar Medical Research Institute), Barcelona (Spain)

^3^Department of Medicine and Life Sciences (MELIS), Universitat Pompeu Fabra Barcelona, 08002 Barcelona, Spain

^4^CIBER of Infectious Diseases

Institute of Health Carlos III, 28029 Madrid, (Spain)

^5^ Emergency Department, Hospital del Mar, Barcelona (Spain)

^6^ Pathology Department, Hospital del Mar, Barcelona (Spain)

^7^Immunexpress - Seattle (United States)

* shared senior authorship

^§^ Corresponding author. Email address: [bbellosillo@psmar.cat](mailto:bbellosillo@psmar.cat)

Running title: SeptiCyte RAPID for COVID-19 triage

**Supplementary Material**

**Supplementary Table S1**  **Clinical data capture (from Case Report Form)**

| Demographics (age, sex, ethnicity/race) |
| --- |
| SARS-CoV-2 testing results (confirmed SARS-CoV-2 positive status by RT-qPCR or antigen test) |
| Clinical variables and medical history^a^ |
| Vital signs^b^ (values collected in the first 24 hours after hospital admission) |
| Laboratory variables^c^ (values collected in the first 24 hours after hospital admission, unless noted otherwise) |
| Microbiology tests (during hospital stay) (Performed yes/no; findings) |
| Serology tests (during hospital stay) (Performed yes/no; findings) |
| CBC w/differential (WBC, lymphocytes, neutrophils, platelets, bands), during hospital stay |
| Other viral tests, during hospital stay (Performed yes/no; findings) |
| Radiologic evaluation/imaging results |
| Hospital (and ICU) length of stay |
| Therapeutic treatments/interventions (antivirals, antibacterials, corticosteroids, vasopressors, inotropes, supplemental O2, mechanical ventilation, ECMO, RRT/dialysis) |
| Final diagnosis as per care team |
| Mortality data |
| ^a^ Presence of chronic infection, presence and description of comorbidities, presence of immunosuppression.  ^b^ Temperature, respiratory rate, heart rate, mean arterial pressure, systolic blood pressure (highest, lowest)  ^c^ CRP, D-dimer, IL6, lactate, procalcitonin, oxygen saturation (within the first 72 hours). |

**Supplementary Table S2 Clinical interventions provided to the patients stratified by COVID severity.** Numbers in parentheses ( ) are percentages.

| **Interventions** | **Mild** | **Moderate** | **Severe** | **Critical** | **Critical & Died** |
| --- | --- | --- | --- | --- | --- |
|  | **(n=10)** | **(n=27)** | **(n=70)** | **(n=39)** | **(n=13)** |
| Antivirals | 0 | 2 (7.4) | 19 (27) | 12 (31) | 4 (31) |
| Antibacterials | 4 (40) | 8 (30) | 34 (49) | 34 (87) | 11 (85) |
| Steroids | 3 (30) | 20 (74) | 70 (100) | 39 (100) | 13 (100) |
| Vasopressors | 0 | 0 | 1 (1.4) | 18 (46) | 8 (62) |
| Inotropes | 0 | 0 | 3 (4.3) | 19 (49) | 10 (77) |
| Low flow O_2_ | 4 *(40) | 16 (59) | 67 (96) | 33 (85) | 7 (54) |
| High flow O_2_ | 0 | 2 (7.4) | 40 (57) | 39 (100) | 13 (100) |
| Mechanical ventilation (MV) | 0 | 1 (3.7) | 0 | 17 (44) | 5 (38) |
| MV + intubation | 0 | 0 | 0 | 18 (46) | 9 (69) |
| ECMO | 0 | 0 | 0 | 1 (2.6) | 1 (7.7) |
| RRT / dialysis | 2 (20) | 0 | 1 (1.4) | 4 (10) | 3 (23) |
| ICU admission | 0 | 0 | 9 (13) | 31 (79) | 9 (69) |
| Death | 0 | 0 | 0 | 13 (33) | 13 (100) |

***low flow O2 was given for patient comfort**

***\***

**Supplementary Table S3 Variables used in the Principal Component Analysis The variables were of two types:**

**(1) Quantitative variables defining the eigenvectors used to construct the PCA dimensions; and (2) Supplementary continuous variables not used to construct the PCA dimensions The R package FactoMinerR [Le et al., 2008] was used for the PCA.**

| **No.** | **Variables for PCA analysis** | **Type of variable** |
| --- | --- | --- |
| 1 | SeptiCyte.Score | Quantitative |
| 2 | Temperature max | Quantitative |
| 3 | Heart Rate min | Quantitative |
| 4 | Arterial Pressure min Systolic | Quantitative |
| 5 | Percent lymphocytes | Quantitative |
| 6 | Percent neutrophils | Quantitative |
| 7 | neutrophils lymphocytes ratio | Quantitative |
| 8 | high flow O2 total days event1 | Quantitative |
| 9 | high flow O2 total days event1+2 - combining the number of days a patient was on high flow oxygen during event 1 and event 2 | Quantitative |
| 10 | mechanical ventilation total days event 1+2 - combining the number of days a patient was on mechanical ventilation during event 1 and event 2 | Quantitative |
| 11 | Creatinine | Quantitative |
| 12 | Procalcitonin (PCT) | Quantitative |
| 13 | Systolic Blood Pressure max | Quantitative |
| 14 | O2 saturation first 72 hours min | Quantitative |
| 15 | MAP min | Quantitative |
| 16 | WBC.count | Quantitative |
| 17 | HA lowO2 event1 start | Quantitative |
| 18 | Age | Quantitative |
| 19 | Temperature min | Quantitative |
| 20 | Arterial Pressure max Systolic | Quantitative |
| 21 | Arterial Pressure min Diastolic | Quantitative |
| 22 | low flow O2 total days event1 | Quantitative |
| 23 | high flow O2 total days event2 | Quantitative |
| 24 | low flow O2 total days event1+2 - combining the number of days a patient was on low flow oxygenation during events 1 and 2 | Quantitative |
| 25 | intubation with MV total days event 1 2 - combining the number of days a patient was on intubation with mechanical ventilation during events 1 and 2 of the intervention. | Quantitative |
| 26 | Lactate | Quantitative |
| 27 | Respiratory Rate max | Quantitative |
| 28 | Systolic Blood Pressure min | Quantitative |
| 29 | D-dimer | Quantitative |
| 30 | Platelets | Quantitative |
| 31 | HA highO2 event1 start - Days between hospital admission (HA) and the start of high flow oxygenation event 1 | Quantitative |
| 32 | Hospital total stay days | Quantitative |
| 33 | Heart Rate max | Quantitative |
| 34 | Arterial Pressure max Diastolic | Quantitative |
| 35 | low flow O2 total days event2 | Quantitative |
| 36 | PAXgene collection from hospital admission days | Quantitative |
| 37 | high flow O2 total days event 1+2+3+4+5 (combining the number of days a patient was on high flow oxygen, during all separate intervals of O2 administration | Quantitative |
| 38 | IL-6 | Quantitative |
| 39 | C-reactive protein (CRP) | Quantitative |
| 40 | Respiratory Rate min | Quantitative |
| 41 | O2 saturation first 72 hours max | Quantitative |
| 42 | MAP max | Quantitative |
| 43 | cells percent sum | Quantitative |
| 44 | SpO2/FiO2 ratio calculated for the partial SOFA score | Quantitative |
| 45 | total days on O2 therapy - combining total number of days on either high or low flow oxygenation | Quantitative |
| 46 | qSOFA partial score | Supplementary continuous |
| 47 | SOFA partial score | Supplementary continuous |
| 48 | Number of SIRS criteria | Supplementary continuous |
| 49 | Sex | Supplementary continuous |
| 50 | Presence of immunosuppression | Supplementary continuous |
| 51 | Death | Supplementary continuous |
| 52 | COPD - Chronic Obstructive Pulmonary Disease | Supplementary continuous |
| 53 | Cancer | Supplementary continuous |
| 54 | CKD - Chronic Kidney Disease | Supplementary continuous |
| 55 | DIC - Disseminated Intravascular Coagulation | Supplementary continuous |
| 56 | Diabetes | Supplementary continuous |
| 57 | Dyslipidemia | Supplementary continuous |
| 58 | Smoking | Supplementary continuous |
| 59 | CVD - Cardiovascular Disease | Supplementary continuous |
| 60 | Other viral tests during hospital stay | Supplementary continuous |
| 61 | Antibacterials | Supplementary continuous |
| 62 | Inotropes | Supplementary continuous |
| 63 | Intubation with Mechanical Ventilation | Supplementary continuous |
| 64 | Mechanical ventilation | Supplementary continuous |
| 65 | Race | Supplementary continuous |
| 66 | Presence of chronic infection | Supplementary continuous |
| 67 | Microbiology tests during hospital stay | Supplementary continuous |
| 68 | Steroids | Supplementary continuous |
| 69 | ECMO - Extracorporeal Membrane oxygenation | Supplementary continuous |
| 70 | Low flow Oxygenation required? | Supplementary continuous |
| 71 | SeptiCyte RAPID Interpretation Band | Supplementary continuous |
| 72 | Presence of comorbidities | Supplementary continuous |
| 73 | Serology tests during hospital stay | Supplementary continuous |
| 74 | Antivirals | Supplementary continuous |
| 75 | Vasopressors | Supplementary continuous |
| 76 | RRT – Renal Replacement Therapy /dialysis | Supplementary continuous |
| 77 | High flow Oxygenation required? | Supplementary continuous |
| 78 | Hypertension | Supplementary continuous |
| 79 | Obesity | Supplementary continuous |
| 80 | CLD - Chronic Liver Disease | Supplementary continuous |
| 81 | CT scan done? | Supplementary continuous |

**Supplementary Figure S1 Principal Component Analysis of Complete Cohort The two groups within the study cohort that differed to the greatest extent were: (red) critical and severe COVID cases that needed ICU admission (N=40), and (black) mild and moderate cases that did not need ICU admission (N=37). In addition, (green) the remaining 69 patients in the cohort, who were adjudicated as severe or critical COVID cases but not admitted to ICU, were also used.**

**
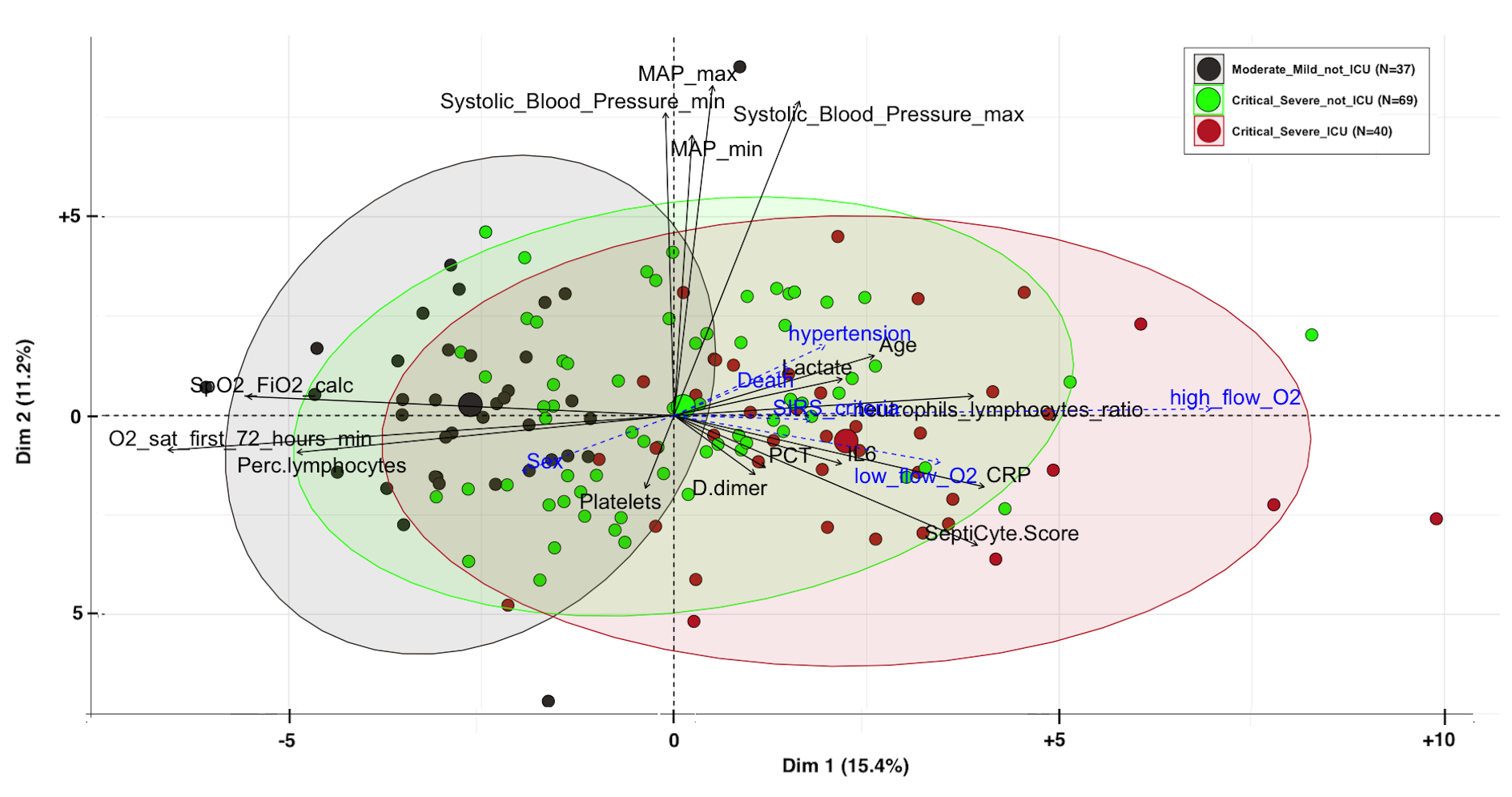
**
